# Effects of Temperature Increase on Microbiome of Carnivorous Plant *Utricularia vulgaris* L. in Peat Bog Ecosystems

**DOI:** 10.3390/biology14070884

**Published:** 2025-07-18

**Authors:** Aleksandra Bartkowska-Bekasiewicz, Tomasz Mieczan

**Affiliations:** Department of Hydrobiology and Protection of Ecosystems, University of Life Sciences, Dobrzańskiego 37, 20-262 Lublin, Poland

**Keywords:** microorganisms, traps, response, resilience, temperature

## Abstract

Climate change can have a direct impact on the decomposition of organic matter, as well as indirect effects on peatland vegetation (including carnivorous plants) and the microbial communities associated with this environment. The activity of microbes varies depending on the type of peatland they inhabit. Because some microorganisms are highly sensitive, they can be used as indicators of climate change. However, there is still little knowledge of how changes in the temperature of the environment can affect the microbiome of carnivorous plants. The study was conducted to test the following hypotheses: (1) The effect of rising water temperature on the qualitative and quantitative structure of the microbiome of carnivorous peatland plants depends on the type of peatland; (2) habitats with a higher trophic status stimulate the development of microbial communities in the water, but are an unfavourable habitat for the development of the microbiome of plant traps. Irrespective of the type of peatland, the species richness of microorganisms was much higher in the water than in the traps. As the temperature increased, there was an increase in the abundance of bacteria, heterotrophic flagellates, and testate amoebae, which was much more pronounced in the peat bog than in the carbonate fen.

## Simple Summary

Climate change significantly affects peatland ecosystems, which are among the most sensitive and endangered in Europe. Carnivorous plants, such as *Utricularia vulgaris*, together with their associated microbial communities, are integral components of these habitats. This study aimed to investigate how increasing water temperature influences the abundance, biomass, and diversity of microorganisms living both in the water and inside plant traps, in two contrasting peatland types: a *Sphagnum* peat bog and a carbonate-rich fen. The research demonstrated that microbial communities are more abundant and diverse in water than in plant traps. Elevated temperatures generally stimulated microbial growth, especially in habitats with a higher trophic status; however, such conditions negatively affected the microbiome within the plant traps. The results suggest that the microbiome of *Utricularia vulgaris* traps reflects the fertility of the surrounding environment. These findings are crucial for understanding how global warming may alter the delicate balance of peatland ecosystems and their biodiversity. This knowledge is valuable for future conservation efforts, as it identifies early bioindicators of climate change and informs strategies to protect vulnerable wetland species and their habitats.

## 1. Introduction

Global climate change is a significant intercontinental problem. It is mainly the result of intensive hydrological changes (caused by an unnatural increase in temperature), which have a significant impact on terrestrial ecosystems [1]. As early as 2007, the Intergovernmental Panel on Climate Change (IPCC) presented data on a possible temperature increase of 2–4 °C within the next few decades [2,3]. According to the latest report, climate change models are changing faster than predicted [4,5]. The temperature increase will be accompanied by extreme weather phenomena, such as droughts and heat waves [6]. Global warming will adversely affect boreal forests, tundras, and peatlands [7]. A growing number of studies indicate that peatland ecosystems will be especially vulnerable to climate change [8,9,10]. This is particularly concerning, as peatland ecosystems are among the most endangered ecosystems in Europe [11]. Wetlands accumulating peat occupy only 3% of global land area and store about 30% of organic carbon [12,13]. Climate change, as well as human activity, can decrease the amount of C in the environment [14,15]. According to prognoses, the carbon balance may undergo highly significant fluctuations in 2020–2100 [16].

Peatlands are also inhabited by endangered plants, including many species of carnivorous plants [17]. Disturbances caused by climate change lead to changes in the species composition of vegetation and the microorganisms associated with it, which leads to the faster degradation of these areas. For this reason, peatlands are increasingly becoming model ecosystems in studies of biodiversity and the effect of global warming on the functioning of ecosystems [18]. There have been few studies investigating the relationships between an increase in temperature and differences in the trophic state of peatlands. Studies on climate change or variable concentrations of biogenic compounds have been carried out in specific types of peatlands—usually raised bogs [19,20,21,22].

According to the literature data, many endangered plant species may have difficulty adapting to changing climatic conditions. A study by [23] showed that nearly 70% of species have a negative median bioclimatic velocity, and some species may completely lose their habitats by 2050 [1]. Recent studies have shown that this also applies to protected species closely associated with peatland ecosystems [24,25,26,27]. These include carnivorous plants, whose means of obtaining resources, i.e., predation, has allowed them to survive in difficult habitat conditions (low levels of minerals, low pH, and a low oxygen concentration in the soil) [28,29,30]. Carnivorous peatland plants attract, capture, and kill animal organisms in order to obtain food by absorbing their biomass [1]. Plants of the genus *Pinguicula* secrete sticky mucous substances to capture insects. Plants of the genera *Utricularia* and *Genlisea* use modified leaves that form highly specialized traps. *Utricularia* develops active suction (bladder) traps, which are triggered by the mechanical stimulation of sensory hairs. When the trap is activated, it rapidly opens and generates a vacuum that draws in small organisms, including microorganisms such as bacteria and protozoa, which may be free-living or attached to particles [28,29,30]. In contrast, *Genlisea* forms passive (lobster-pot) traps, where prey enter through a spiral channel and are guided toward the digestive chamber by inward-pointing hairs that prevent escape. Once inside either trap type, the captured organisms are digested by plant-secreted enzymes [31,32,33].

Microbial communities are closely associated with carnivorous peatland plants. They can be present in the traps as well as on the surface of the plant. Adamec, Płachno et al., and Sirovà et al. demonstrated the presence of microorganisms such as cyanobacteria, bacteria, and ciliates in the traps of carnivorous plants [34,35,36]. Microorganisms and small Metazoa are highly sensitive to changes in environmental conditions, so they are increasingly used in experimental models. Knowledge of the effects of climate change on microbial communities in different types of peatlands is crucial to determining the impact of global warming on these ecosystems. Previous studies have focused on determining the response of individual species or populations to increases in temperature in a single habitat (a single type of peatland) [37,38,39,40,41,42,43].

The literature data indicate that some microbial communities living in peatlands can be early indicators of disturbances in peatland ecosystems [44]. Most studies have presented analyses of the size of microbial communities, without considering changes in their biomass, which provide important information about the ecosystem [11]. There have also been no studies investigating observable differences in the microbiome of carnivorous plants inhabiting raised bogs, transitional bogs, and fens in the case of experimental modelling of temperature. The previous literature results have shown that microbial communities are not indifferent to temperature changes [11]. Research by Mieczan and Tarkowska-Kukuryk (2015) and by Mieczan and Bartkowska (2022) showed an increase in the abundance of bacteria, heterotrophic flagellates, and ciliates in conditions of elevated temperature [43,45]. An increase in temperature positively affects the abundance of bacteria and heterotrophic flagellates and the biomass of testate amoebae and ciliates in water [46]. However, there is little information on the microbial communities colonizing the traps of carnivorous peatland plants. Higher temperature has been shown to increase the mortality of microorganisms in traps [35,36,47]. This may be associated with a change in habitat fertility, as increased nitrogen concentrations in the environment reduce the plant’s reliance on acquiring microbial prey through its traps to supplement nutrient deficiencies [48]. In the present study, an attempt was made to compare the responses of the microbial communities present in plant traps and in the surrounding water to climate change (temperature change) depending on the type of peatland (peat bog vs. carbonate fen). The main purpose of the study was to conduct an in-depth analysis of the impact of changes in temperature on the microbiome of traps of *Utricularia vulgaris* L.—a carnivorous plant inhabiting various types of peatlands. The laboratory experiment served to verify the following research hypotheses:

1. The effect of rising water temperature change on the qualitative and quantitative structure of the microbiome of carnivorous peatland plants depends on the type of peatland.

2. Habitats with a higher trophic status (carbonate fen vs. peat bog) stimulate the development and multiplication of microbial communities in the water but are an unfavourable habitat for the development of the microbiomes of plant traps.

## 2. Materials and Methods

### 2.1. Experiment and Laboratory Analyses

**Figure 1:**
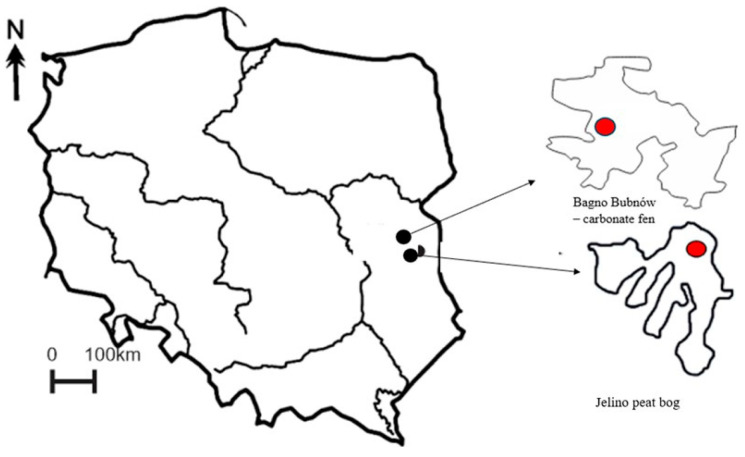
Study area.

An experiment was carried out in laboratory conditions to determine the effect of an experimental temperature change on the microbiome of *Utricularia vulgaris* L. The temperature in the experiment was raised by 2 °C, 4 °C, and 8 °C relative to the control treatment. The samples for the experiment were obtained from two types of peatland: the Jelino transitional bog with elements of a raised bog—J (eastern Poland, 51°24.009′ N, 23°9.116′ E) and the Bagno Bubnów carbonate fen—BB (51°22.364′ N, 23°15.303116′ E) (Figure 1). Water was collected directly from the surface layer of each peatland site using a sterile 1 L glass bottle. Samples were taken from the central part of each site to avoid edge effects, at approximately 5–10 cm depth to capture the representative water column.

The samples were transported to the laboratory and stored for two days in the same glass containers that were later used for the experimental treatments. These containers had a volume of 500 mL. This approach was chosen to minimize sample handling and potential disturbance, ensuring consistency between the storage and experimental conditions. To ensure homogeneity, the collected water was thoroughly mixed in a sterile glass container before being divided into experimental units. Mixing was performed gently but thoroughly using a sterilized glass rod for approximately 5 min to prevent the settling of microorganisms.

**Figure 2:**
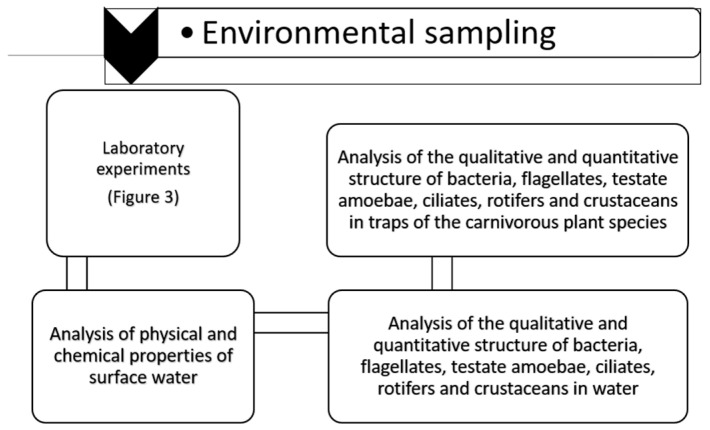
Design of the experiment.

**Figure 3:**
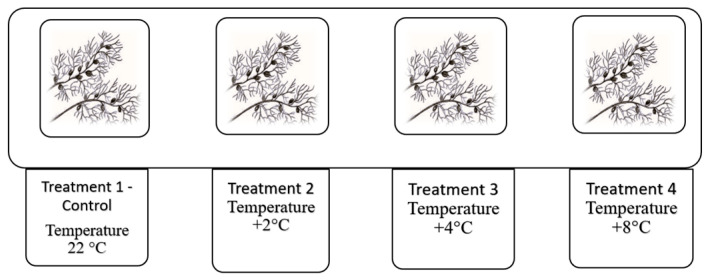
Design of the laboratory experiment in four temperature treatments.

The samples were kept for two days in glass containers with a 16 h/8 h light/dark regime to stabilize the environmental conditions. Then the experiment was carried out in four treatments: treatment 1—control (C), treatment 2—temperature increased by 2 °C relative to the control, treatment 3—temperature increased by 4 °C relative to the control, and treatment 4—temperature increased by 8 °C relative to the control. The experimental treatments were carried out in triplicate. *Utricularia vulgaris* L. plants were placed in 500 mL glass containers filled with water from the peatlands (five plants in each). Then the temperature was manipulated using an electric heating device (Figure 2 and Figure 3).

Samples for biological and physicochemical analysis were collected at the start of the experiment and after 21 days. To assess the effect of the habitat on the microbiome of *Utricularia*, samples were collected from each treatment for qualitative and quantitative analysis from the water surrounding *Ultricularia vulgaris* L. (250 mL) and from the traps. A water sample and five traps were collected from each experimental treatment. Five traps per treatment were randomly selected at the end of the exposure period. Each trap was carefully removed using sterile tweezers to avoid cross-contamination, then immediately placed into individual sterile Petri dishes or glass vials for further microscopic and taxonomic analysis. A volume was gently taken using a sterile pipette or syringe from the mid-layer of the water column to avoid disturbing sediment or floating matter. Two samples were preserved with Lugol’s solution (2% concentration), and one live sample was taken (from both the water and the traps). The abundance and biomass of the bacteria, heterotrophic flagellates, testate amoebae, ciliates, rotifers and crustaceans were determined. Abundance and biomass of bacteria were determined using DAPI (4′,6-diamidino-2-phenylindole)-staining, according to Porter and Feig (1980); abundance and biomass of flagellates was also assessed by using DAPI-staining (according to Caron, 1983) [49,50]; and abundance and species composition of testate amoebae, ciliates, and crustaceans according to Utermöhl [51]. In the case of rotifers, 50 mL samples were placed in a cylinder sealed with parafilm for 24 h, after which the upper 5 mL was removed. To determine abundance and biomass, three samples were preserved in Lugol’s solution and analyzed using plankton chambers with an inverted microscope (Nikon, Type 104c, Nikon Corporation, Tokyo, Japan). For DAPI-stained samples, we used a Nikon epifluorescence microscope equipped with a UV filter set, at 1000× magnification (oil immersion). For live samples and settled material analyzed according to the Utermöhl method, we used a Nikon inverted microscope, working primarily at 100× and 400× magnification depending on the organism group observed. The abundances and species compositions of testate amoebae, ciliates, and crustaceans were determined from the same settled subsample of each experimental replicate to ensure consistency in taxonomic and abundance comparisons. For each group (testate amoebae, ciliates, and crustaceans), a minimum of 100 individuals per group per sample was counted whenever possible. If a group was less abundant, all observable individuals within the settled volume were included.

The traps for analysis were placed in Petri dishes, washed with water filtered through a filter with 0.2 µm pore size, and examined under a microscope. After removal from the treatments, traps were carefully placed in sterile 90 mm Petri dishes containing a small amount (3–5 mL) of treatment water to preserve moisture and prevent sample degradation. The abundance of organisms attached to or present inside the traps was determined using a Nikon stereomicroscope at 40× to 100× magnification. For smaller organisms and a detailed morphological observation, a Nikon compound microscope was used at 200× to 1000× magnification (with oil immersion when needed). Abundance was quantified by either scanning the entire trap surface (in the case of low-density samples) or by analyzing randomly selected fields of view and extrapolating to the full trap area. Biomass of microorganisms was determined using the following conversion factors: heterotrophic bacteria 1 µm^3^ = 5.6 × 10^−7^ µgC; flagellates 1 µm^3^ = 2.2 × 10^−7^ µgC; and ciliates and testate amoebae 1 µm^3^ = 1.1 × 10^−7^ µgC [52]. Biomass of rotifers was determined based on the ratio of the body length to the body weight of the specimen [53]. The plants were not deposited in the herbarium after the research was completed.

The physical and chemical parameters of the water were analyzed as well: temperature, electrical conductivity, pH, biogenic compounds, chemical oxygen demand (COD), biological oxygen demand (BOD), chlorophyll *a* and total organic carbon (TOC), total suspended solids (TSS), and surfactants. Temperature, electrical conductivity, and pH were determined with a multiparameter probe (YSI 556 MES). Total organic carbon (TOC), total nitrogen, total phosphorus, and biological and chemical oxygen demand were analyzed by spectrophotometry (UV-VIS Spetrophotometer; ThermoFisher Scientific, Waltham, MA, USA).

### 2.2. Statistical Analysis

Statistical analysis was carried out to determine the effects of the physical and chemical parameters of the water on the abundance of microbial communities. First, a detrended correspondence analysis (DCA) was performed to calculate the gradient of variation representing the data, which determined the type of analysis applied. In addition, the Monte Carlo permutation test was performed to identify the environmental variables which statistically significantly (*p* < 0.05) influence the abundance of microorganisms. The analyses were carried out in CANOCO 5.0 and Statistica 13 software. The significance of differences between means was determined by a one-way analysis of variance (ANOVA). Tukey’s test was used to determine which groups differed significantly. The relationships between environmental parameters and organismal groups (bacteria, flagellates, ciliates, testate amoebae, and crustacea) were analyzed using a redundancy analysis (RDA). Prior to RDA, both biological and environmental data were standardized to equalize their scales and to reduce the influence of variables with large value ranges. Biological abundance data and environmental variables were appropriately pre-processed to ensure statistical validity and comparability. The abundance of organismal groups was log-transformed using the function log(x + 1). Prior to analysis, correlation coefficients between environmental variables were examined. In cases of high collinearity, one of the correlated variables was excluded from further analysis.

All data generated or analyzed during this study are included in this published article in a variety of format. Moreover, the datasets generated during and/or analyzed during the current study in different formats are available from the corresponding author.

## 3. Results

### 3.1. Environmental Variables

Analysis of the physical and chemical parameters of the water showed that the increase in temperature in the peat bog was accompanied by increases in pH, electrical conductivity, chlorophyll a, and TSS. The highest pH (6.96) was noted at the temperature 2 °C higher than in the control, while the highest electrical conductivity (43.4 μS cm^−1^), chlorophyll a (83 mg L^−1^), and TSS (180 mg L^−1^) were recorded at the highest temperature (+8 °C). In the carbonate fen, the physical and chemical responses to increased temperature varied by treatment. TSS increased from 294 mg/L in the control to a peak of 315 mg/L in variant II. TOC values showed a marginal increase from 41.0 mg/L in the control to 42.0 mg/L in variant III. Notably, conductivity decreased slightly in variants II and III (120.9 and 118.5 µS cm^−1^, respectively) before increasing sharply to 176.1 µS cm^−1^ in variant IV. Minor increases in surfactants and BOD were also observed across treatments (*p* < 0.05 for all, Table 1).

### 3.2. Species Diversity and Abundance of Microorganism and Small Metazoa

#### 3.2.1. Abundance of Bacteria

**Figure 4:**
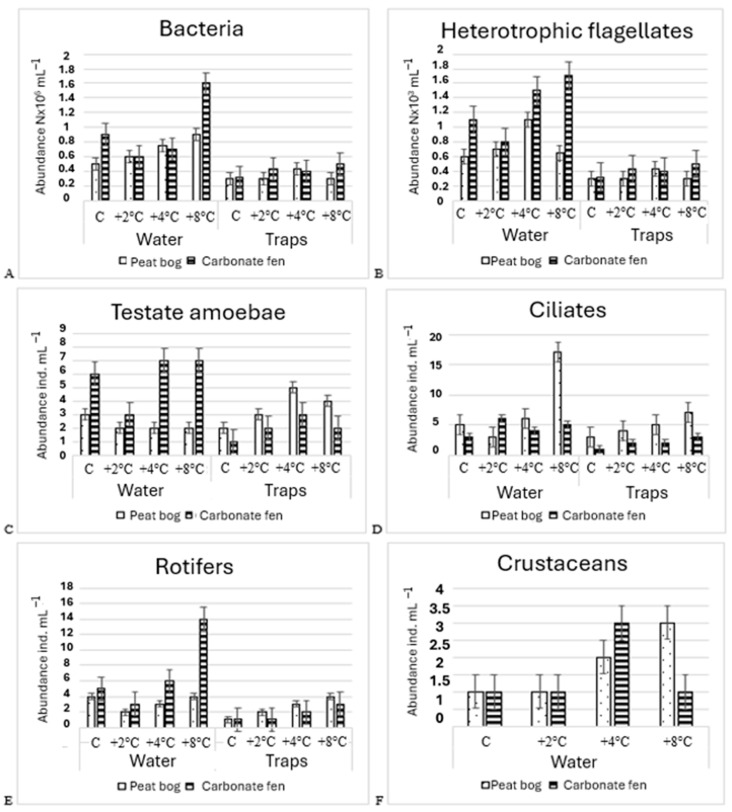
Changes in the abundance of (**A**) heterotrophic bacteria, (**B**) heterotrophic flagellates, (**C**) testate amoebae, (**D**) ciliates, (**E**) rotifers, and (**F**) crustaceans in the water and carnivorous plant traps in each experimental treatment (in different types of peatland) (C—control; ± SD—standard deviation).

The abundance of bacteria was markedly higher in the water than in the traps of *Utricularia vulgaris* L. (in both the peat bog and the carbonate fen) in comparison to the control treatment. The abundance of bacteria in the water was markedly higher in the carbonate fen (0.61–1.59 × 10^6^ cells mL^−1^) than in the *Sphagnum* peat bog (0.51–0.92 × 10^6^ cells mL^−1^). Bacterial abundance in the water was generally higher in the carbonate fen than in the *Sphagnum* peat bog across most treatments; however, at +4 °C, a higher bacterial count was observed in the peat bog, while values at +2 °C were nearly identical in both systems. In the traps, abundance was, in most cases, higher in BB (0.3–0.5 × 10^6^ cells mL^−1^) than in the peat bog, in which it was in the range of 0.3–0.45 × 10^6^ cells mL^−1^. The highest abundance in the water was recorded in the fourth experimental treatment (+8 °C) in both types of peatlands (ANOVA F_3.30_ = 0.667; *p* = 0.502; Figure 4A).

#### 3.2.2. Abundance of Heterotrophic Falgellates

In the water and in the traps from the carbonate fen, the abundance of flagellates tended to increase with the temperature, although these differences were not statistically significant (*p* > 0.05), including between the +2 °C and +4 °C treatments. The highest abundance in the water was 1.65 × 10^3^ cells mL^−1^, while the highest number in the traps was 0.66 × 10^3^ cells mL^−1^ (ANOVA F_3.30_ = 0.775; *p* = 0.512). In the *Sphagnum* peat bog, the abundance of flagellates was in the range of 0.6–1.15 × 10^3^ cells mL^−1^ in the water and 0.27–0.4 × 10^3^ cells mL^−1^ in the traps (ANOVA F_3.30_ = 0.853; Figure 4B).

#### 3.2.3. Species Diversity and Abundance of Testate Amoebae

Species diversity did not differ significantly between experimental treatments in either peatland. However, there were significant differences in the species diversity between the two types of peatland. In the water from the peat bog in the control treatment, there were four species of testate amoebae, while there were three species in the traps. In the treatments with elevated temperature, the number of species was lower than in the control (from two to three in the water and from one to two in the traps).

In the peat bog, the abundance of testate amoebae in the water reached its highest recorded value in control (3 ind. mL^−1^), although this was only slightly higher than at +2 °C, +4 °C, and +8 °C. In the traps, the highest abundance was observed at +4 °C (5 ind. mL^−1^). In the water from the carbonate fen, the highest abundance was recorded in the third and fourth experimental treatment (+4 °C and +8 °C; 7 ind. mL^−1^), while in the traps, it was highest in the third experimental treatment (+4 °C; 3 ind. mL^−1^) (Figure 4C).

The dominant species in the water from the peat bog in the control treatment were *Arcella discoides*, *Amphitrema flavum*, and *Hyalosphenia papilio*, while *Arcella discoides* was the most numerous species in the traps. In the carbonate fen, the *dominant species were Arcella discoides* and *Euglypha* in the water and *Arcella discoides* in the traps.

#### 3.2.4. Species Diversity and Abundance of Ciliates

The increase in temperature in the experimental treatments caused an increase in the species diversity of ciliates, in both the *Sphagnum* bog and the carbonate fen (*p* < 0.05). In the water from the peat bog, from 10 to 16 ciliate taxa were recorded in the treatments with elevated temperature, as compared to seven taxa in the control. The control traps contained two ciliate taxa, while in each of the treatments with elevated temperature, they contained four taxa. In the water sample from the carbonate fen, there were eight ciliate taxa in the treatments with elevated temperature and four in the control. In the traps, three ciliate taxa were noted in the treatments with higher temperature and two in the control.

The abundance of ciliates differed significantly depending on the habitat and experimental treatment. In the traps from both the *Sphagnum* bog and the carbonate fen, the abundance of ciliates was highest in experimental treatment 4 (+8 °C). In the water from the peat bog, abundance was highest in the last experimental treatment (+8 °C), while in the carbonate fen, it was highest in the second treatment (+2 °C) (Figure 4D). Ciliates were more abundant in the water than in the traps (they show a tendency) (ANOVA F_3.30_ = 0.877; *p* < 0.05).

The dominant ciliate species in the water from the peat bog were *Paramecium bursaria*, *Heliophrya rotunda*, and *Litonotus lamella*, while *Paramecium bursaria* was the most numerous species in the traps. In the carbonate fen, *Paramecium putrinum* and *Stentor* sp. were dominant in the water, while *Paramecium putrinum* was most abundant in the traps.

#### 3.2.5. Species Diversity and Abundance of Small Metazoa

The number of species of rotifers and crustaceans was higher in the peat bog than in the carbonate fen. Rotifers were present in the water and traps of both types of peatland, although their species diversity was shown to decrease as the temperature increased. Crustaceans were only present in the water, with more species recorded in the bog than in the carbonate fen (three taxa vs. one taxon).

The abundance of rotifers (8 ind. mL^−1^) was also higher in the carbonate fen than in the peat bog (2 ind. mL^−1^) (ANOVA F_3.30_ = 0.633; *p* < 0.05). In the traps from the peat bog, the abundance of rotifers was higher (3 ind. mL^−1^) than in the carbonate fen (2 ind. mL^−1^) (Figure 4E,F).

The dominant rotifers in the peat bog were *Bdelloidea* and *Lecane*, while *Bdelloidea* was dominant in the traps. Among crustaceans, the dominant taxon was *Ceriodaphnia* sp. In the carbonate fen, *Bdelloidea* was dominant in both the water and the traps.

### 3.3. Biomass of Microbial Communities

In the carbonate fen, the highest bacterial biomass in the water ranged from 0.6 µg C mL^−1^ (in the +8 °C experimental treatment) to 1.1 µg C mL^−1^ (in the fourth experimental treatment). The highest biomass of heterotrophic flagellates was also observed in the fourth experimental treatment (+8 °C). Testate amoebae, ciliates, rotifers, and crustaceans (in the water and in the traps) attained the highest biomass in treatment 3 (testate amoebae 0.8 µg C mL^−1^ in the water; 0.3 µg C mL^−1^ in the traps; ciliates 0.7 µg C mL^−1^ water; 0.6 µg C mL^−1^ traps; rotifers 0.9 µg C mL^−1^ water; 0.6 µg C mL^−1^ traps; and crustaceans 0.48 µg C mL^−1^ water; Table 2). The biomass of the microbial communities in the peat bog was lower than in the carbonate fen. The biomass of bacteria in the water was highest in the second experimental treatment (+2 °C), while in the traps it was highest in the control. In all other cases (flagellates, testate amoebae, and ciliates), the highest biomass was attained at a temperature +4 °C higher than the control temperature (Table 2).

### 3.4. Size Structure, Correlations, and Ordination Analysis

#### Size Structure and Correlations Between Food Web Components

**Figure 5:**
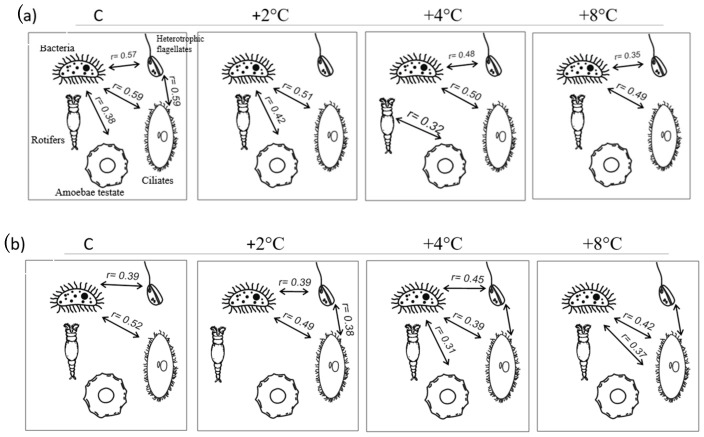
Linear correlation coefficients between food web elements in the water; (**a**) peat bog, (**b**) carbonate fen.

The degree of correlation between microbial communities was varied. In the water, significantly higher correlations were obtained in the peat bog. In the control treatment, bacteria were correlated with heterotrophic flagellates, ciliates, and amoebae (r = 0.57; r = 0.59; and r = 0.38), heterotrophic flagellates with ciliates (r = 0.59), and testate amoebae only with bacteria (r = 0.38). As the temperature increased, the only constant relationship was the correlation between bacteria and ciliates (C: r = 0.59; +2 °C: r = 0.50; +4 °C: r = 0.51; and +8 °C: r = 0.49). In the control treatment from the carbonate fen, there were positive correlations between bacteria and heterotrophic flagellates (r = 0.39) and between bacteria and ciliates (r = 0.52). At a temperature raised 2 °C relative to the control, there was also a correlation between heterotrophic flagellates and ciliates (r = 0.38). In the third experimental treatment (+4 °C) there were significant correlations between bacteria and heterotrophic flagellates (r = 0.45), between bacteria and ciliates (r = 0.39), and between bacteria and testate amoebae (r = 0.31). In the fourth experimental treatment (+8 °C), significant correlations were obtained only between bacteria and ciliates (r = 0.37) and between heterotrophic flagellates and ciliates (r = 0.42) (Figure 5; *p* ≤ 0.05 for all).

**Figure 6:**
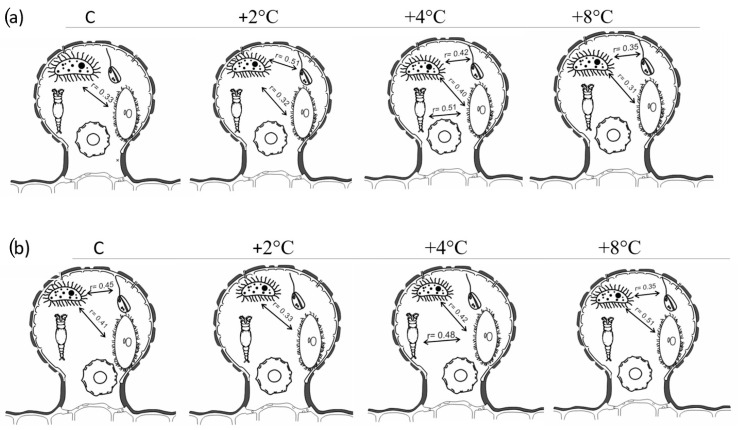
Linear correlation coefficients between food web elements in carnivorous plant traps; (**a**) peat bog, (**b**) carbonate fen.

In the traps from the peat bog, the abundance of bacteria in the control treatment was positively correlated with ciliates (r = 0.43). In the second (+2 °C) and fourth (+8 °C) experimental treatments, the abundance of bacteria was positively correlated with ciliates (+2 °C: r = 0.49; +4 °C: r = 0.42). In the third experimental treatment (+4°), there were significant correlations between bacteria and heterotrophic flagellates (r = 0.42), between bacteria and ciliates (r = 0.4), and between ciliates and rotifers (r = 0.51). In the carbonate fen, in the first (C) and fourth (+8°) experimental treatments, the abundance of bacteria was positively correlated with that of heterotrophic flagellates and ciliates. In the second experimental treatment (+2 °C), a correlation was observed between bacteria and ciliates (r = 0.33), while in the third experimental treatment (+4 °C), there were significant correlations between bacteria and heterotrophic flagellates (r = 0.45) (Figure 6; *p* ≤ 0.05 for all).

### 3.5. Ordination Analysis

**Figure 7:**
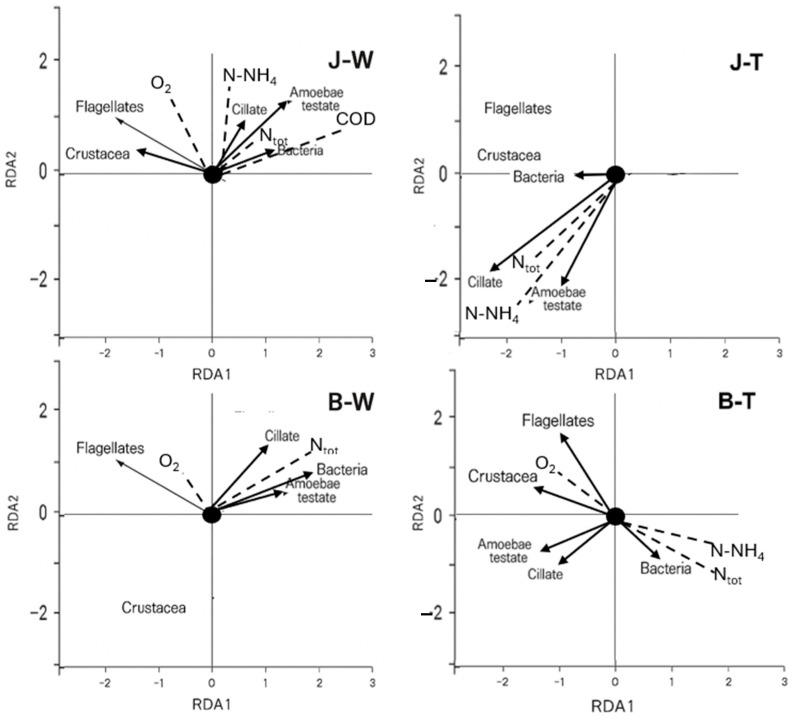
Redundancy analysis biplots showing microbial communities and Metazoa and environmental variables; (J—peat bog, B—carbonate fen, W—water, and T—traps; biological vectors are indicated by bold solid lines and environmental vectors by dashed lines).

Environmental variables were standardized prior to the RDA. The results showed that N-NH_4_, total nitrogen (Ntot), and oxygen were the key factors shaping microbial communities in both the peat bog and the carbonate fen. In the peat bog, microbial communities in the water were primarily influenced by oxygen and nitrogen concentrations, while in the traps, nitrogen concentration was the main driver. In the carbonate fen, microbial communities in the water were shaped by N-NH_4_ and Ntot, whereas in the traps, both nitrogen and oxygen played a major role (Figure 7).

**Figure 8:**
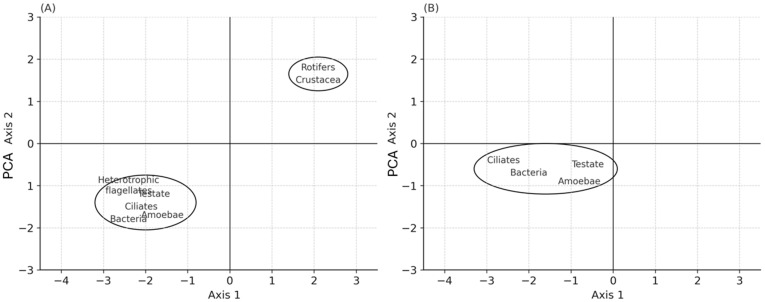
PCA presenting microbial communities in the experimental treatments: (**A**) peat bog; (**B**) carbonate fen.

The results of PCA revealed a division of the microbial communities into two groups in the peat bog and one main group in the carbonate fen. In the peat bog, the first group included bacteria, heterotrophic flagellates, testate amoebae, and ciliates, and the second contained rotifers and crustaceans. PCA showed one main group in the carbonate fen, consisting of bacteria, testate amoebae, and ciliates (Figure 8).

## 4. Discussion

The determination of the consequences of global warming and its effect on peatland ecosystems in particular is extremely important [54,55,56,57]. Climate change can affect not only vegetation but also microbial communities, which play a key role in the functioning of peatland ecosystems [58,59,60]. The response of microbial communities to changes in environmental conditions depends on the type of peatland as well as on the extent of the changes in abiotic parameters [61]. Le-Geay et al. (2024) analyzed the impact of climate change on bacteria present in peatland ecosystems [62]. The literature data indicate that bacteria react dynamically to global temperature changes. In the present study, as the temperature increased, the abundance of bacteria increased in both the peat bog and the carbonate fen. An increase in bacterial abundance accompanying higher temperature was also observed by Le-Geay et al. [62]. The present study showed a higher abundance of bacteria in the carbonate fen, with a high trophic status and high electrical conductivity. This may suggest that a higher trophic state may favour the development of bacteria. Moreover, higher temperature has an indirect positive effect on the trophic status of the habitat, which may be responsible for the increase in the abundance of bacteria in the carbonate fen. Le-Geay et al. (2024) showed that both an increase in temperature and drought can reduce the abundance of bacteria [62]. This is also confirmed by research by Jiang et al. [63]. Studies by Strakova et al. (2011), Urbanov and Bárt (2016), Mpamah et al. (2017), and Potter et al. (2017) also indicate that bacteria are highly sensitive to changes in climate [63,64,65,66,67]. As not only abundance can be an indicator of climate change, attention should also be devoted to changes in the biomass of microorganisms [44]. The present study showed an increase in bacterial biomass in the experimental treatments in comparison with the control. Similar findings have been reported by Adllasing et al. (2011), Giang et al. (2015), and Mieczan and Bartkowska (2022) [32,43,68].

The increase in temperature was also accompanied by an increase in the abundance of flagellates. Irrespective of the experimental treatment, the abundance of these microorganisms was higher in the water than in the traps. Similar results were obtained by Mieczan and Bartkowska, who also observed an increase in the abundance of flagellates accompanying an increase in temperature. This is surprising, given that in the summer the abundance of these microorganisms often decreases due to the increase in predation pressure from rotifers and crustaceans [42,69]. This was observed by Mieczan and Tarkowska-Kukuryk, who reported that the density of heterotrophic flagellates showed a marked decrease when the temperature increased by 4 °C and 8 °C [43,45]. However, that study was conducted only in *Sphagnum*-dominated microhabitats. An increase in temperature can indirectly increase habitat fertility. Therefore, the occurrence of flagellates seems to be influenced by the temperature and trophic status of the habitat as well as by the presence of predators controlling the abundance of these microorganisms.

Peatlands are characterized by a high diversity of microhabitats containing many species of testate amoebae. Due to their specific ecological preferences, they are regarded as reliable indicators of environmental conditions [70]. The morphological and physiological traits of testate amoebae have been shown to be associated with various environmental variables in wetlands [71,72,73,74,75]. The number of taxa is controlled by variable habitat conditions. Water availability (e.g., water table depth) has often been identified as the most important factor controlling the composition of communities of testate amoebae in peatlands, with pH and electrical conductivity as the second most important variables. The results of the present study indicate that pH and electrical conductivity are conducive to the biodiversity of testate amoebae. In addition, in the peat bog, a reduction was shown in the abundance of testate amoebae in the experimental treatments compared to the control. In the carbonate fen, however, their abundance was increased in comparison to the control. Lower density of testate amoebae accompanying elevated temperature has also been observed by Jassey et al. (2015) and by Basińska et al. (2020) [11,76]. This was most likely due to the lower abundance of bacteria and heterotrophic flagellates than in the carbonate fen.

Wilken et al. (2013) and Jessey et al. showed that an increase in temperature leads to a reduction in mixotrophic testate amoebae [76,77]. However, according to Magnan et al. (2018), global climate change can lower the water level in peatlands, which is conducive to the development of *Sphagnum*, whose presence has a positive impact on the occurrence of mixotrophic testate amoebae [78]. On the other hand, an increase in habitat temperature and fertility can result in changes in the biomass and species structure of vegetation. *Sphagnum* gives way to shrubs, whose woody structure is not so easily decomposed [79,80]. Nevertheless, future climate change can also influence the sequestration of C in peatlands through changes in the species structure and biomass of vegetation.

In the present study, the increase in temperature caused a reduction in the number of species of testate amoebae in both the peat bog and the carbonate fen. The reduction in species diversity was more significant in the carbonate fen. Jessey et al. showed a significant relationship between the number of testate amoebae species and the concentration of biogenic compounds [76,77]. As the concentration of nutrient compounds increased, the number of species of these organisms decreased. Similar relationships were noted in the present study. It is likely that due to the higher trophic status of the carbonate fen, it had a lower species diversity of testate amoebae than the transitional bog. The dominant species in the peat bog were *Arcella discoides*, *Amphitrema flavum*, and *Hyalosphenia papilio. In the carbonate fen, the dominant species in the water were Arcella discoides and Euglypha sp*. These are mixotrophic amoebae which colonize wet habitats (with a high water level) [74,75,81,82]. Climate change, defined in part by rising temperatures, can cause stress and fluctuations in environmental parameters, leading to changes in the structure and functioning of peatland ecosystems, which can also influence the biodiversity of testate amoebae [83]. Mixotrophic testate amoebae obtain the majority of carbon during photosynthesis, and thus mainly function as autotrophs [84]. A higher abundance of these microorganisms is also conducive to carbon accumulation, but this process can vary depending on environmental conditions (moisture and temperature) [85].

The present study also showed a negative effect of increased temperature on the biomass of testate amoebae. Basińska et al. (2020) reported that even a small manipulation of the temperature in the natural environment caused a reduction in the biomass of testate amoebae [11]. Similar results were obtained by Jassey et al. (2011), Lamentowicz et al. (2013), Koenig et al. (2017), and Reczuga et al. (2018) [44,86,87]. In the present study, one of the dominant species in the peat bog was *Hyalosphenia papilio, which is the main predator feeding on ciliates* [88]. Basińska et al. (2020) showed that mixotrophic testate amoebae (such as *Hyalosphenia papilo*) can be regarded as early indicators of climate change [11]. Mixotrophic microorganisms, including amoebae, exhibit a high rate of biomass change and are highly sensitive to increases in environmental temperature [89,90]. Jessey et al. found that a long-term increase in the temperature of the environment (from +2 to +8 °C) can lead to a reduction in the biomass of the dominant mixotrophic testate amoebae. Lower abundance and biomass of testate amoebae may suggest the presence of predators for which testate amoebae are a food base [91,92]. The present study showed a reduction in the abundance and biomass of testate amoebae (in the peat bog) or only abundance (in the carbonate fen), accompanied by an increase in the abundance and species richness of ciliates relative to the control. Importantly, the abundance of ciliates differed depending on the experimental treatment. Studies by Mitchell et al., Kexin et al., Nguyen-Viet et al., and Wilkinson and Mitchell have shown that ciliates are common in high numbers in peatland ecosystems [91,92,93]. Lukic et al. (2022) showed that higher temperatures are favourable to an increase in the abundance of ciliates [94]. Furthermore, according to the literature data, ciliates are extremely sensitive to changes in habitat conditions due to their thin cell membrane. For this reason, their reaction time to environmental changes is much shorter than in the case of other microorganisms [95].

In the present study, from 10 to 16 ciliate taxa were present in the peat bog water in the treatments with elevated temperature, as compared to seven taxa in the control. In the carbonate fen, there were eight ciliate taxa in the treatments with elevated temperature and four in the control. The dominant ciliate species in the water from the bog were *Paramecium bursaria*, *Heliphrya rotunda*, and *Litonotus lamella*, while *Paramecium bursaria* was the most abundant species in the traps. *Paramecium putrinum* and *Stentor* sp. were the dominant taxa in the water from the carbonate fen, and *Paramecium putrinum* was the most abundant species in the traps. Mieczan and Bartkowska (2022) reported that as the temperature increased there was an increase in the number of ciliate species. There were 12–19 taxa in the water (the dominant species were *Paramecium bursaria*, *Urocentrum turbo*, and *Litonotus lamella*) and four in the traps (with a dominance of *Paramecium bursaria*). However, these analyses only apply to *Sphagnum*-dominated peatlands. There are no comparative data on ciliates associated with carnivorous plants in carbonate fens.

Rotifers play an especially important role in the food chain. They are good indicators of changes in the environment due to their rapid reaction to changes in environmental conditions and stress factors [96]. In the present study, rotifers were clearly dominant among small Metazoa. The abundance of these organisms was higher in the carbonate fen. The higher habitat fertility and higher temperature contributed to an increase in the abundance of bacteria and heterotrophic flagellates, which are food for this group of organisms [97]. The abundance of rotifers decreased as the temperature increased (in J and BB). Pociecha et al. (2023) also compared the response of rotifers in varied climate conditions (the effects of reduced and elevated temperature) [98]. However, that study was conducted only in raised bogs. This was probably the reason for the significant decrease in the abundance of these organisms following the temperature manipulation. Temperature was a stress factor which negatively affected their abundance. It can be concluded that rotifers prefer stable environmental conditions, and temperature changes can result in a decrease in the abundance of small Metazoa.

The number of rotifer species was higher in the peat bog than in the carbonate fen. Their species diversity was shown to decrease as the temperature increased. *Bdelloidea* and *Lecane* were dominant among rotifers in the *Sphagnum* bog, and *Bdelloidea* was also dominant in the carbonate fen. The genus *Lecane* is very common in peatland ecosystems [99]. It can also be an indicator of climate change, as it has been shown to occur in much higher numbers in water bodies with higher temperatures than in water bodies in the temperate climate zone [97].

The degree of colonization of traps by microorganisms depends not only on environmental factors such as temperature and trophic status but also on the age of the traps and the presence of zooplankton. Younger traps generally contain fewer microorganisms, whereas older, more mature traps accumulate a greater number of prey and decomposing organic matter, which promotes the colonization by bacteria and other microorganisms [100]. Additionally, the presence of zooplankton in the environment can influence the composition of the trap microbiome by serving as a source of colonizing organisms and participating in local trophic interactions. Adamec, Płachno and Sirovà have demonstrated the presence of microorganisms such as bacteria in the traps of carnivorous plants. In the present study, the abundance of these microorganisms was higher in the water than in the traps. As bacteria are the main source for heterotrophic flagellates, the increase in bacterial abundances with raised temperatures may also have caused the increase in this group in plant traps. For the same reason (predation of flagellates on bacteria) the comparable lower number in the plant traps vs. the water may be a consequence of intense predation by flagellates. Buosi et al. (2011) also showed that the abundance of bacteria increases with temperature, and that it is higher in the water than in plant traps [100]. The literature data indicate that bacteria are able to survive and multiply inside carnivorous plant traps [101]. In addition, they can take part in the digestion of prey or can themselves constitute a food base for other organisms [102]. Traps operate less intensively in an environment with a high trophic status, which probably explains why the abundance of bacteria was higher in the peat bog than in the carbonate fen [32].

Heterotrophic flagellates are a relatively stable element of the microbiome of *Utricularia vulgaris* L. [103]. There is little information on the functioning of this group in carnivorous plant traps. The present study showed an increase in the abundance of flagellates as the temperature increased. Their highest biomass was recorded in the control treatment, and the lowest at a temperature 8 °C higher than in the control. According to the literature data, carnivorous plant traps are sensitive to changes in environmental conditions. The increase in temperature positively influenced the abundance of heterotrophic flagellates but negatively affected their biomass. Their abundance may have been controlled by testate amoebae, which are more numerous in the water than in traps. Heterotrophic flagellates and bacteria were a potential food source for amoebae; moreover, owing to their high abundance, they did not need to compete with other microorganisms for resources [11]. It can be concluded that amoebae play a key role as predators in the structure of the microbial food web. This is supported by the results of the present study, in which the biomass of testate amoebae was markedly higher in the treatments with elevated temperature than in the control (as the temperature increases, the abundance of flagellates and bacteria constituting a food source for testate amoebae increases as well). In addition, the species composition of testate amoebae is highly sensitive to changes in environmental conditions. This is confirmed by the present study and research by Mitchell et al. and Marcisz et al. In the present study, *Arcella discoides* was the dominant species in the traps in both the peat bog and the carbonate fen. This species has a broad ecological tolerance and is often present in nutrient-rich habitats. The high trophic state of the carbonate fen was probably the reason for the dominance of *Arcella discoides* [76].

Not only testate amoebae can influence the abundance of bacteria and flagellates; ciliates can also function as their consumers. Ciliates exert a significant effect on the structure and dynamics of prokaryotes. They can also constitute a food base for small Metazoa. The present study showed fewer ciliate species in the traps than in the water. However, an increase in temperature is known to result in a higher metabolic rate, which in turn increases the energy requirements of microorganisms. Thus, an increase in temperature and high habitat fertility should create a suitable living environment for ciliates. On the other hand, increased fertility negatively affects the biodiversity of these microorganisms, which was confirmed in the present study (there were fewer species in the carbonate fen than in the peat bog). Research by DeWitt et al. (2024) [103] indicated that low nutrient concentrations have a greater impact than temperature. It can be concluded that an increase in fertility and temperature adversely affects ciliates in traps, in contrast to their effect on these microorganisms in the water [103]. The trophic network exerts a substantial influence on the functioning of peatland ecosystems. Ciliates may function as primary consumers of bacteria and heterotrophic flagellates, while simultaneously serving as a key food source for small Metazoans. Additionally, they are capable of exhibiting intraguild predation by feeding on other ciliates. Furthermore, ciliates may constitute an important trophic resource for higher-level consumers, such as rotifers [81].

Rotifers feed on bacteria, heterotrophic flagellates, and protozoans. However, they may feed on other rotifers as well. They can also constitute a food base for testate amoebae. It is likely that the high abundance of testate amoebae regulated the abundance of rotifers, which resulted in their low abundance in the traps. It can be concluded that ciliates—microorganisms constituting potential resources for rotifers—did not undergo significant predation pressure, which may explain their high abundance in the experimental treatments.

## 5. Conclusions

The obtained research results are also particularly significant due to the fact that the material acquired for analysis was small, which presents a certain kind of experimental limitation. The study made it possible to verify the research hypotheses. Climate change was shown to significantly influence the qualitative and quantitative structure of the microbiome of carnivorous plants in peatlands. The species richness and abundance of microorganisms and small Metazoa were much higher in the water than in the plant traps, in both the *Sphagnum* peat bog and the carbonate fen. However, the impact of climate change varies depending on the type of habitat. A higher trophic state is favourable to microbial communities in the water but unfavourable to the microbiome of plant traps. The microbiome of *Utricularia vulgaris* L. traps is a reflection of the trophic state of the surrounding environment. The higher the concentration of biogenic compounds in the habitat, the less work is required of the traps, which results in a lower abundance of microorganisms in these habitats.

The analyses provide a substantial contribution to the knowledge of the impact of climate change on the microbiome of carnivorous plants. This is particularly important given increasing global climate change and the extreme sensitivity of peatland ecosystems to these changes. The present study is another step towards a better understanding of the critical transformations taking place in peatland ecosystems associated with the changing climate. Future studies should take into account the effect of the increasingly frequent increases in the abundance of cyanobacteria on microbial communities in wetland habitats. Moreover, future studies could also attempt to consider the formation of resting stages in protozoans as a result of thermal stress.

## Figures and Tables

**Table 1:** Changes in physical and chemical parameters in four experimental mesocosms in different types of peatbogs.

Peatland				Carbonate Fen				
	Variant I C = 22°	Variant II +2 °C	Variant III +4 °C	Variant IV +8 °C	Variant I C = 22°	Variant II +2 °C	Variant III +4 °C	Variant IV +8 °C
**T (°C)**	22.5	24.6	25.6	30.1	22.3	24.6	25.6	30.4
**pH**	4.98	6.96	6.9	6.91	6.51	5.9	6.4	6.44
**Cond. (μS cm^−1^)**	27.1	31	29.2	43.4	147.4	120.9	118.5	176.1
**O_2_ (mg L^−1^)**	7.83	8.09	7.77	7.06	6.12	7.72	7.98	4.06
**O_2_%**	94.2	97.6	95.3	89.4	73.7	93.6	98.8	52.4
**N_tot_ (mg L^−1^)**	1.843	1.203	1.397	1.418	2.987	3.05	3.270	4.444
**P_tot_ (mg L^−1^)**	0.124	0.071	0.01	0.046	0.131	0.06	0.02	0.209
**Chl_a_ (µg L^−1^)**	24	20	31	83	25.32	26	6.32	23
**SUR (mg L^−1^)**	23	22.4	22	22.8	34.5	35.5	36	35
**ChZT (mg L^−1^)**	68.5	65.6	65.5	68.8	85	85	86	85
**BZT (mg L^−1^)**	41.5	39.5	39.5	41	53	54	54.5	53.5
**TSS (mg L^−1^)**	156	176	168	180	294	315	300	305
**TOC (mg L^−1^)**	31.5	30	30	31	41	41.5	42	41.5

**Table 2:** The biomass of microbial communities in different experimental variants—in different types of peatlands.

.	Average Level of Biomass (µgC mg L^−1^)															
	Peatland								Carbonate Fen							
Environment 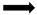	Water				Traps				Water				Traps			
Taxonomic group 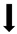																
	C	+2 °C	+4 °C	+8 °C	C	+2 °C	+4 °C	+8 °C	C	+2 °C	+4 °C	+8 °C	C	+2 °C	+4 °C	+8 °C
Bacteria	0.52	0.72	0.83	0.91	0.42	0.47	0.49	0.53	0.6	0.82	0.97	1.1	0.5	0.55	0.62	0.65
Heterotrophic falgellates	0.2	0.45	0.62	0.75	0.19	0.42	0.47	0.51	0.32	0.55	0.71	0.82	0.2	0.4	0.52	0.5
Testate amoebae	0.5	0.6	0.7	0.4	0.1	0.1	0.2	0.1	0.6	0.6	0.8	0.5	0.1	0.2	0.3	0.2
Ciliates	0.3	0.3	0.8	0.6	0.2	0.2	0.6	0.4	0.4	0.5	0.7	0.6	0.3	0.2	0.7	0.5
Rotifers	0.7	0.8	0.4	0.3	0.6	0.5	0.2	0.1	0.8	0.8	0.9	0.4	0.5	0.5	0.6	0.2
Crustacea	0.6	0.45	0.38	0.3	-	-	-	-	0.70	0.52	0.48	0.39	-	-	-	-

## Data Availability

The original contributions presented in this study are included in the article. Further inquiries can be directed to the corresponding author(s).
